# Uncovering a new SASH1 mutation associated with dyschromatosis universalis hereditaria using whole-exome-sequencing: A case report

**DOI:** 10.1097/MD.0000000000034448

**Published:** 2023-08-04

**Authors:** Yue Yang, Nan Jiang, Jing Qun Mai, Shuo Yang, Yuanyuan Xiao, Shanling Liu

**Affiliations:** a Department of Obstetrics and Gynecology, West China Second Hospital of Sichuan University, Chengdu, China; b Key Laboratory of Birth Defects and Related Diseases of Women and Children, Sichuan University, Ministry of Education, Chengdu, China; c State Key Laboratory of Oral Diseases and National Clinical Research Center for Oral Diseases, West China Hospital of Stomatology, Sichuan University, Chengdu, China; d Department of Liver Surgery, State Key Laboratory of Biotherapy and Cancer Center, West China Hospital, Sichuan University and Collaborative Innovation Center of Biotherapy, Chengdu, China; e West China School of Medicine, Sichuan University, Chengdu, Sichuan, China.

**Keywords:** case report, dyschromatosis universalis hereditarian, missense mutation, *SASH1*, whole-exome sequencing

## Abstract

Dyschromatosis universalis hereditaria (DUH) is an uncommon form of pigmented genodermatosis that is typically inherited autosomally and dominantly. In the previous study, the pathogenic genes of DUH have been identified in ATP-binding cassette subfamily B, member 6 and *SASH1*. However, the mutational screening of the causative gene remains incomplete and still lacks sufficient proof in the etiology.

**Patient concerns:**

A 2-generation Chinese family clinically diagnosed with DUH were enrolled. They showed pigmented spots from their childhood and came to the hospital for medical advice and genetic analysis. We found a novel mutation c.1757T > C (p.I586T) of *SASH1* in 3 affected family members by whole-exome sequencing.

**Diagnoses:**

Genetic outcomes and clinical examinations confirmed the diagnosis of DUH in 3 family members with lentiginous syndrome.

**Interventions and outcomes:**

Using whole-exome sequencing and sanger sequencing technologies, we identified a novel mutation c.1757T > C (p.I586T) of *SASH1* that co-segregated in 3 afflicted family members but not in the normal individuals. Significantly, c.1757T > C (p.I586T) is a novel mutation which had not been previously reported. The same codon position in *SASH1* (c.1758C > G, p.I586M) has been reported in a Japanese man, and he showed identical phenotype compared to our study participants.

**Lessons:**

Our study broadens the spectrum of DUH mutations and provides more genetic characteristics of DUH in understanding its etiology. Furthermore, we demonstrated the diagnostic accuracy of whole-exome sequencing for inherited skin diseases and provided new information for etiological study.

## 1. Introduction

The genetically heterogeneous pigmentary genodermatosis, known as dyschromatosis universalis hereditarian (DUH), is featured by hyper- and hypopigmented macules in reticulate pattern that develop within the childhood stage of the patient and affect almost the whole body.^[1]^ Some cases have reported the accompanied systemic change occurrence, such as deafness,^[2]^ visual impairment,^[3]^ and neurological symptoms.^[4]^

As the hereditary disorder, modes of inheritance that are both autosomal dominant and autosomal recessive have been confirmed in DUH.^[5,6]^ The DUH-related genes were initially identified on chromosomes 6q24.2 to q25.2 and 12q21 to q23.^[6,7]^ In 2013, Zhang^[8]^ further confirmed the disease-causing mutations to be in the ATP-binding cassette subfamily B, member 6 gene at 2q35 in patients diagnosed with DUH. Hence, according to its various chromosomes connecting regions, DUH can be divided into 3 types: DUH1(OMIM 127500) located in the 6q24.2 to q25.2, DUH2 (OMIM 612715) located in the 12q21q23 and DUH3 (OMIM 615402) located in the 2q35. Novel findings also identified *SASH1* as related genes to DUH.^[9]^ Therefore, determining the pathogenic genes is of vital importance for developing diagnostic tools and potential therapies for DUH.

In this report, we present the discovery of finding a novel *SASH1* missense mutation in southwestern China in 2 DUH-diagnosed generations. The family members with DUH symptoms showed unequal-sized pigmented spots since their childhood and came to hospital for genetic analysis and further treatment. Besides, we also reviewed the published literatures of *SASH1* related pigmentation abnormalities. As further exploration of the genetics of DUH is being conducted, we aim to provide more information about the genetic characteristics of DUH and the application of gene therapy in clinical settings for it.

## 2. Methods

### 2.1. Clinical findings and diagnosis

**Figure 1.**
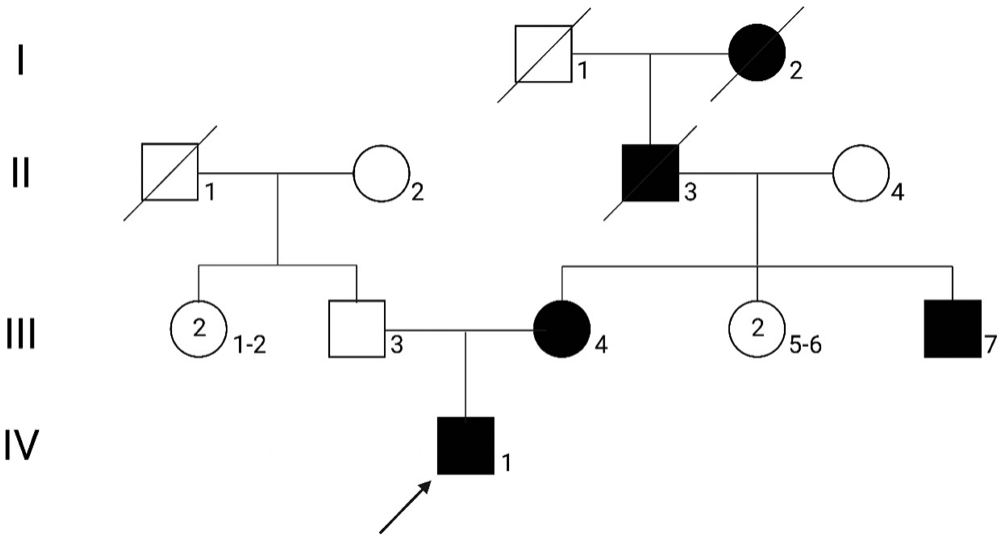
The DUH family pedigree chart. The WES-proband is marked with an arrow. Squares and circles are used to represent males and females, respectively. Black-filled symbols indicate family members with symptoms of pigmentation. DUH = dyschromatosis universalis hereditarian, WES = whole-exome sequencing.

To confirm the clinical diagnosis, each affected member underwent meticulous physical examinations and thorough reviews of their hospital medical records. We investigated the patient and his family members who were diagnosed with DUH and exhibited signs of autosomal dominant pigmentary disorder. Figure 1 depicts the family genogram map, with IV1 serving as the whole-exome sequencing-proband (WES-proband).

**Figure 2.**
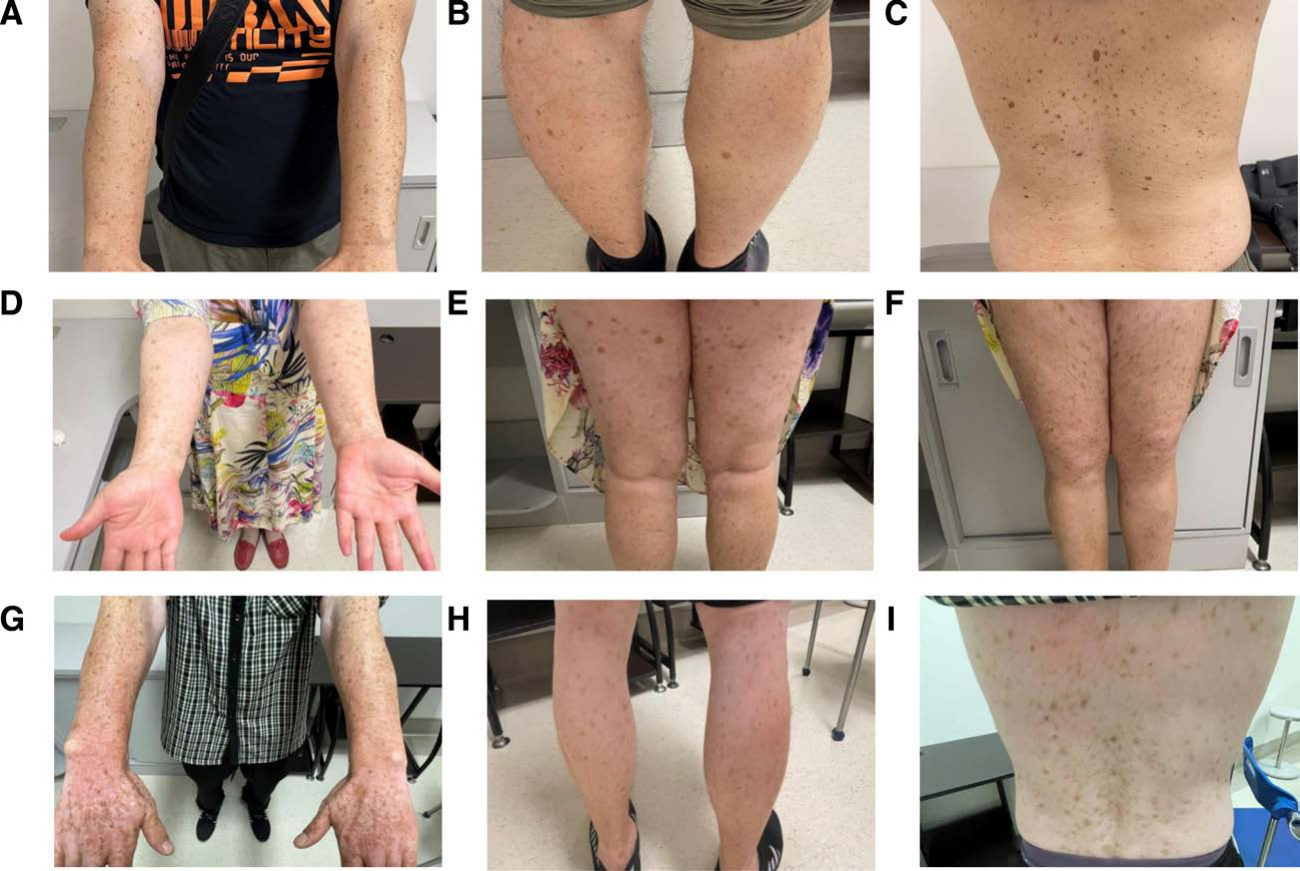
(A–C) Mottled hypo- and hyper-pigmented macules on the trunk and limbs in the WES-proband. (D–F) Cutaneous manifestations of the WES-proband’s mother. Generalized hyperpigmentation spots mixed with hypopigmentation spots in a reticular pattern. (G–I)Cutaneous manifestations of the WES-proband’s uncle. Freckled pigmentation diffusely on his limbs and trunk. The pigmentation is more severely distributed on his arms. WES = whole-exome sequencing.

The individual who served as the WES-proband was a 30-year-old Chinese young man with lesions of differing size and color over his limbs and trunks. When he was 1 year old, these lesions initially emerged on his cheek as lentigines and then steadily spread to his limbs, face, and neck over the years, while the face and trunk showed graver symptoms. He was diagnosed with multiple lentigines and was treated with laser on his face afterwards. Clinical examination showed mild hyper-and depigmented macules of varying sizes on his face while a large amount of macules on his limbs and trunk (Fig. 2A–C).

Similar skin lesions were observed on the whole body of the patient’s mother (III4), uncle (III7), late grandfather (II3), and late great-grandmother (I2), suggesting a potential autosomal dominant mode of inheritance. On the WES-proband’s mother’s limbs, there was a smattering of pigmentation with light brown color. (Fig. 2D–F). The lesions that were seen on the limbs and back of the WES-proband’s uncle were more representative, with clearly and reticular arranged widespread hyperpigmentation areas alternating with hypopigmentation spots (Fig. 2G–I).

### 2.2. Whole-exome sequencing

The family members’ peripheral blood leukocytes were taken, and total DNA extraction was obtained using the QIAamp DNA Blood Mini Kit (QIAGEN, Hilden, Germany) in conformity with the manufacturer’s protocol. Three people were chosen to complete the trio-whole-exome sequencing: the WES-proband, his mother, and his father (Trio-WES). To detect both the concentration and degree of purity of the total extracted DNA, we use a NanoDrop 2000 UV–vis spectrophotometer (Termo Fisher Scientific, Massachusetts, United States). To establish the gene types of the patients, we utilized the system Nano WES Human Exome V1 (Berry Genomics) to collect the sequences. Besides, a Nova seq 6000 was used to sequence a total of 150 paired-end reads from the enriched library. The Burrows-Wheeler Aligner was employed to align the gene sequencing reads to the human reference genome (UCSC GRCh38/hg38). Afterwards, Verita Trekker (v1.2.0.2) was performed to test the new variation.

All variants were filtered by various publicly available data resources, which include the Genome Aggregation Database (gnomAD, http://gnomad.broadinstitute.org/), the Exome Aggregation Consortium (ExAC, http://exac.broadinstitute.org/), and the 1000 Genomes Database (https://www.internationalgenome.org/). Only minor allele frequency thresholds 0.05 or lower were used to identify variants. The qualified variants were defined as rare populations, and were preserved for further research. In silico tools including Sorting Intolerant From Tolerant (SIFT, https://sift.bii.a-star.edu.sg/), Mutation Taster (http://www.mutationtaster.org/), and Polymorphism Phenotyping v2 (Polyphen-2, http://genetics.bwh.harvard.edu/pph2/) were employed to analyze the potential impact of each variant on the structure and function of the protein.

To access whether the amino acid substitution was conservative or radical, conservative analysis was carried out as described in Homologene (https://www.ncbi.nlm.nih.gov/homologene). Next, SWISS-MODEL (https://swissmodel.expasy.org) was applied to simulate how amino acid substitutions influence the 3-dimensional conformation of proteins. After that, variations that were identified as pathogenic were checked against the human gene mutation database (HGMD, http://portal.biobaseinternational.com/ghmd/pro/search_gene.php) and ClinVar database (http://www.ncbi.nlm.nih.gov/clinvar) to see if they were already known or if they were new. The nomenclature scheme utilized for naming the newly discovered gene variants adheres to the international standards set forth by the Human Genome Variant Society, as outlined on their website (http://www.hgvs.org/mutnomen).

Following the examination of all probable variants, the pathogenicity of the detected sequence variations was assessed in accordance with the American College of Medical Genetics and Genomics (ACMG) guidelines^[10]^ and Expert Specification of the and the ACMG/Association for Molecular Pathology Variant interpretation Guidelines for genodermatosis.

### 2.3. Sanger sequencing

Subsequently, in order to confirm the *SASH1* alterations, the WES-proband and his family members were subjected to Sanger sequencing. Using the gene tool programme, primers were created to amplify particular areas with variations through polymerase chain reaction. Through a genetic analyzer instrument ABl3730, the polymerase chain reaction products were subjected to direct sequencing (Applied Biosystems, California, United States). Chromas software was used to evaluate Sanger sequencing results (Technelysium, South Brisbane, Australia). Furthermore, variations were systematically classified as pathogenic, potentially pathogenic, or of unknown clinical importance using guidelines from the ACMG.

## 3. Results

**Figure 3.**
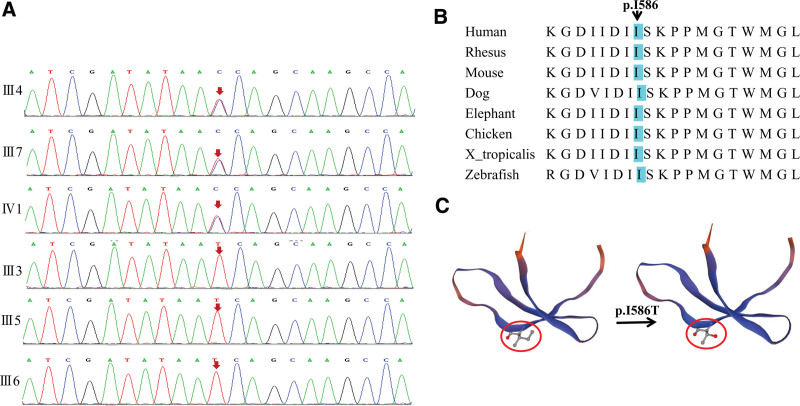
(A) Sanger sequencing chromatograms of III3, III4, III5, III6, III7 and IV1. A missense mutation, c. 1757T > C (p.I586T), in SASH1 was found in the affected family members (III4, III7, and IV1) but not in unaffected members (III3, III5, III6). (B) SASH1 sequence comparison among multiple species showed p.I586 was highly conserved throughout evolution. (C) Model building for conformational changes in amino acids and proteins by SWISS-MODEL.

Based on the Trio-wes outcomes, we discovered a novel heterozygous missense mutation (c.1757T > C, p.I586T) in exon 15 of the *SASH1* (NM_015278) in members of III4, III7, and IV1 (Fig. 3A). While this mutation was not detected in the family members who lacked lentiginous phenotype (III3, III5, and III6). The results of the Sanger sequencing are displayed in Figure 3A. No adverse effects were reported on the participants after examination.

This novel variant has not been included in gnomAD, the Exome Aggregation Consortium (ExAC), or the 1000 Genomes Database. The presence of this novel mutation (c.1757T > C, p.I586T) in *SASH1* results in the substitution of an acid amino in SH3 domain, a highly conserved domain throughout evolution (Fig. 3B). Isoleucine (I) has a side chain and contains an amidogen in the α site, it is hydrophobic and nonpolar. Threonine (T) is polar and hydrophilic. The replacement of amino acids can have important impact on the tertiary structure of proteins, potentially leading to significant changes in functional properties. To confirm the structural alterations in amino acid sequence and polypeptides, protein models were created using the simulation software SWISS-MODEL (Fig. 3C).

Importantly, it should be noted that the same codon position has been reported in a patient with lentiginosis syndromes (c.1758C > G, p.I586M),^[11]^ which has been incorporated into the HGMD. The previously reported case appeared severe lentigines on his face, limbs, and less on his trunk. Comparing the patients who share the same mutation sites, they have a lot of similarities in clinical features. This phenomenon further proved pathogenicities of *SASH1* in pigmentation abnormalities.

As defined by the classifications of the ACMG, the missense mutation (c.1757T > C, p.I586T) is classified as a likely pathogenic variation with 2 moderate (PM2 and PM5), and 2 supporting (PP1 and PP4) pathogenicity evidence.

## 4. Discussion

In this study, we diagnosed a patient, his affected mother, and more affected uncle with DUH. Through Trio-WES and Sanger sequencing, we identified and confirmed a novel mutation (c.1757T > C, p.I586T) in the *SASH1* gene, which was also present in the affected family members.

The *SASH1* gene is located at chromosome 6q24.3 to q25.1, spanning 209,207 base pairs and containing 19 introns and 20 exons. As a member of the SLY family, *SASH1* encodes 2 classic domains: sterile α-motif and SH3 domain-containing protein 1. Initially identified as a tumor suppressor in breast and colon cancers, *SASH1* has since been found to play a critical role in carcinogenesis.^[12,13]^ Downregulation of *SASH1* is associated with aggressive tumor progression, metastasis, and poor prognosis.^[14,15]^ Additionally, the PI3K/Akt and TGF-β1 signaling pathways related to cell migration and invasion are consistent with *SASH1*’s differential expression.^[16,17]^ These findings suggest that *SASH1* may have multiple functions in various cell types.

In recent years, *SASH1* variants have been implicated in different pigmentation disorders, prompting investigations into the role of *SASH1* in dermatosis.^[1,18]^ Studies have confirmed that *SASH1* is a disease-causing locus for DUH and generalized lentiginosis, which typically exhibit an autosomal dominant trait with heterozygous mutations.^[1]^ Research using epidermal tissues from DUH-affected individuals has shown that *SASH1* upregulation promotes melanocyte migration and cell binding.^[9]^ Furthermore, *SASH1* contributes to DUH through regulation of the IQGAP1–E-Cadherin signaling pathway.^[9]^ Later research revealed that mutated *SASH1* alleles can upregulate the p53/POMC signaling cascade, activate the ERK1/2/CREB cascade through p53, and trigger hyperpigmentation.^[19]^ According to the HGMD, a total of 31 disease-causing *SASH1* variants have been reported, linked to various human diseases such as pigmentation disorders, autism spectrum disorder, keratoderma, skin carcinoma, DUH, and multiple lentigines syndrome. Specifically, 4 mutations (4/31, 13.0%) are associated with autism spectrum disorder, while 9 mutations (9/31, 29.0%) lead to DUH. Nine mutations (9/31, 29.0%) are responsible for multiple lentigines syndrome, and 5 mutations (5/31, 16.1%) have been associated with DUH-like pigmentation anomalies, palmoplantar keratoderma, and skin carcinoma, 1 mutation (1/31, 3.2%) is found in developmental disorders. Additionally, 3 mutation sites (3/31, 9.70%) in *SASH1* have been linked to congenital heart disease.

In our study, we employed whole-exome sequencing to successfully identify a novel missense mutation (c.1757T > C, p.I586T) in *SASH1*, located in the SH3 domain, a causative region for lentiginous phenotype. This finding contributes to a better understanding of the genetic underpinnings of DUH and the mechanisms behind the gene. The biological function of the SH3 domain, initially identified in the non-receptor tyrosine kinase Src, is involved in the regulation of tyrosine kinase signaling and enzyme complexes.^[20,21]^ The SH3 domain is primarily responsible for the amino acid change observed in our case. The affected individuals in our study showed similar clinic manifestations, presenting with dark to light brown macules on their limbs, trunk, and face. Interestingly, the same codon position in *SASH1* (c.1758C > G, p.I586M) has been reported in a young Japanese man who exhibited a substantially identical phenotype compared to our study participants. This observation further underscores the relationship between *SASH1* and DUH, and highlights the importance of understanding the gene mechanisms underlying DUH.

## 5. Conclusion

We identified a novel missense mutation (c.1757T > C, p.I586T) in *SASH1* that has not been previously reported. Our study successfully broadens the mutation spectrum of *SASH1*, and proves the diagnostic accuracy of whole-exome sequencing in hereditary skin diseases, which will aid in gene testing, clinical management, and genetic counseling for DUH patients. Moreover, our findings provide more genetic information in understanding the etiology of DUH. To better address DUH and understand its clinical implications, further research on gene therapy for DUH is warranted.

## Author contributions

**Investigation:** Yuanyuan Xiao, Shanlin Liu.

**Methodology:** Jing Qun Mai, Shuo Yang.

**Software:** Yue Yang, Nan Jiang, Jing Qun Mai, Shuo Yang.

**Supervision:** Nan Jiang, Yuanyuan Xiao, Shanling Liu.

**Validation:** Nan Jiang.

**Writing – original draft:** Yue Yang.

**Writing – review & editing:** Yuanyuan Xiao, Shanling Liu.
